# High Prevalence of Human Papillomavirus in Vulvar Cancer Among Vietnamese Women: Implications for Vaccination Strategies

**DOI:** 10.1002/cam4.70982

**Published:** 2025-06-05

**Authors:** D. N. L. Tran, H. N. Vo, T. T. A. Cung, N. D. Tran, H. N. Cao

**Affiliations:** ^1^ Department of Oncology University of Medicine and Pharmacy at Ho Chi Minh City Ho Chi Minh City Vietnam; ^2^ International Ph.D. Program in Medicine, College of Medicine Taipei Medical University Taipei Taiwan; ^3^ Faculty of Public Health University of Medicine and Pharmacy at Ho Chi Minh City Ho Chi Minh City Vietnam; ^4^ Institute Pasteur Ho Chi Minh City Vietnam

**Keywords:** HPV, Vietnam, vulvar cancer, vulvar tumor

## Abstract

**Background:**

Vulvar cancer (VC) is rare; however, its incidence has steadily increased, likely due to increased human papillomavirus (HPV) infections. HPV infection rates vary significantly with age and ethnicity. Data on the VC incidence in Vietnam are limited.

**Objectives:**

This study aimed to determine HPV infection rates and high‐risk HPV types (HR‐HPVs) and to model HPV causality in co‐infections using a Proportional Attribution (PropAttr) model in Vietnamese VC patients.

**Methods:**

We investigated primary VC cases (invasive carcinoma) diagnosed at our hospital during 2020–2021. Tumor samples were tested for HPV using quantitative polymerase chain reaction. Recurrent cases and poor‐quality preserved tumor samples were excluded. HPV infection status, HR‐HPV status, and relevant clinicopathological features were analyzed. To estimate the most likely causative HPV genotype in co‐infected lesions, the PropAttr model was applied, attributing genotypes based on their prevalence in mono‐infections. Model reliability was validated using Spearman's correlation analysis.

**Results:**

Of the 95 cases, 95% were squamous cell carcinoma, and 40% were clinical stage I. The HPV infection rate was approximately 77% (68.4–85.2), and HPV‐16 was the most common subtype. Patients infected with HPV (mean age: 62.8) were younger than those who were not infected (mean age: 71.4) in univariate (*p* = 0.004) and multivariate (*p* < 0.001) analyses. While the vulvar pathohistological type was significantly associated with HPV infection in multivariate analysis (*p* < 0.001), no significant relationship was observed with other factors in univariate and multivariate analyses. The PropAttr model showed significant correlations between attribution estimates and mono‐infection prevalence (HPV‐16: *ρ* = 0.806, *p* < 0.001; HPV‐18: *ρ* = 0.992, *p* < 0.001; 12 other HR‐HPVs: *ρ* = 0.880, *p* < 0.001). A strong negative correlation between HPV‐16 and 12 other HR‐HPVs (*ρ* = −0.751, *p* < 0.001) suggested competitive interactions in genotype assignment.

**Conclusions:**

The HPV infection rate in Vietnamese VC cases was substantially higher than in other Asian populations, indicating a significant public health burden. Our findings reinforce the importance of expanding national HPV vaccination programs and incorporating advanced attribution models to improve HPV‐related cancer risk assessment.

**Abbreviations**
AC: adenocarcinoma
BCC: basal cell carcinoma
CC: cervical cancer
CI: confidence interval
CIN: cervical intraepithelial neoplasia
Cis: cisplatin
COVID: coronavirus disease
CT: computed tomography
dCRT: definitive chemoradiotherapy
EBRT: external beam radiation therapy
FFPE: formalin‐fixed, paraffin‐embedded
FNA: fine‐needle aspiration
GPA: gravida, para, abortus
HCMC: Ho Chi Minh City
HPV: human papillomavirus
HR‐HPVs: high‐risk human papillomavirus types
IVD: in vitro diagnostic
kSCC: keratinizing squamous cell carcinoma
LCM: laser capture microdissection
LN: lymph node
MBC: Molecular Biomedicine Center
nkSCC: non‐keratinizing squamous cell carcinoma
NOS: not otherwise specified
PropAttr: Proportional Attribution
qPCR: quantitative polymerase chain reaction
RLBH: reverse line blot hybridization
SCC: squamous cell carcinoma
SD: standard deviation
UMP: University of Medicine and Pharmacy
VaIN: vaginal intraepithelial neoplasia
VC: vulvar cancer
VIC: vulvar invasive carcinoma
VIN: vulvar intraepithelial neoplasia

## 1 Introduction

Vulvar cancer (VC) is a rare disease, accounting for less than 1% of all cancers among women. According to the Surveillance, Epidemiology, and End Results, the incidence of this disease has increased steadily in young women by 1.3% per year, while the mortality rate has decreased slightly in recent decades [1, 2]. According to the Globocan World (2022), VC is ranked 21st in incidence (0.83 per 100,000 person‐years) and 22nd in mortality (0.3/100,000 person‐years) among women. According to Globocan Vietnam (2022), VC is ranked 24th in incidence (0.28 per 100,000 person‐years) and 25th in mortality (0.1 per 100,000 persons‐year) among women [3].

In 1983, Hausen et al. reported that 99% of all patients with cervical cancer (CC) are infected with human papillomavirus (HPV) [4, 5], fostering an investigation into the pathogenesis [6, 7, 8]. Major advances including HPV vaccination programs and HPV‐DNA‐based screening for CC have led to a significant reduction in the incidence and mortality of CC worldwide [9, 10, 11]. In VC, this rate is approximately 35%–40% of all cases [12, 13, 14]. HPV infection rates vary both geographically (32% in Europe, 45% in Asia, and 57% in North America) and in age (48%, 28%, and 15% in patients aged 15–54, 55–64, and ≥ 65 years old, respectively) [15]. The HPV‐associated pathohistological subtypes include warty squamous cell carcinoma and basal squamous cell carcinoma [12, 13]. Among HPV infections, type 16 predominates (70%–80%), followed by type 33 (< 15%) and type 18 (< 10%) [12, 13]. These findings suggest that VC can be prevented by HPV vaccination, particularly in young Asian women [16].

Regarding pathogenesis, the patient cohort with VC includes two contrasting types of individuals. Individuals with VC infected with HPV are often young (< 45–55), have had many sexual partners, have not been vaccinated against HPV, and have common vulvar intraepithelial neoplasia (VIN) lesions or other HPV‐related primary cancers, such as cancers of the cervix, anal canal, and oropharynx [12]. By contrast, those not infected with HPV are usually older (> 65–75), have several underlying diseases, such as hypertension, diabetes, and metabolic syndrome, and VC is accompanied by atrophic vulvovaginitis, vulvar lichen sclerosus/planus, and differentiated VIN lesions [17]. Moreover, the established “International Society for the Study of Vulvovaginal Disease” terminology for these subtypes, such as “SCC usual” and “SCC warty dysplasia,” was utilized by Gargano et al. [18, 19] Given the molecular pathology of VC, those not infected with HPV often exhibit p53 mutations, while those infected with HPV often express higher levels of p16 [8, 20, 21]. Many studies have shown that women with VC who are infected with HPV have a more favorable prognosis, including better overall survival and disease‐free survival, than the non‐infected, even after multivariate analyses [13, 22, 23].

In Asia, the rate of HPV infection has been widely investigated in gynecological cancers, including VC, which has facilitated comparisons with global data and the establishment of public health policies [24, 25]. In Vietnam, the rate of HPV infection and the association with CC has been extensively investigated; however, minimal studies have researched HPV‐related VC. Moreover, HPV vaccination coverage tends to be low in developing countries for both the 1‐dose regimen and regimens with more doses [26], despite its tangible benefits in CC [9]. Therefore, this study aimed to evaluate the rate of HPV infection and high‐risk HPV types (HR‐HPVs) in patients with VC in Vietnam. Our hope is that the results highlight the importance and benefits of enhancing the country's HPV vaccination coverage.

## 2 Materials and Methods

### 2.1 Study Design

This mixed cross‐sectional study and case report was conducted at the Oncology Hospital and Molecular Biomedicine Center (MBC) of the University of Medicine and Pharmacy (UMP) in Ho Chi Minh City (HCMC). The Oncology Hospital in HCMC, which is affiliated with the UMP, is the main hospital specializing in oncology in southern Vietnam.

### 2.2 Participants

The study cohort comprised patients with VC diagnosed at the Oncology Hospital between January 1, 2020, and December 31, 2021. The inclusion criteria were patients whose cancer pathology was vulvar invasive carcinoma (VIC) and whose tissue samples had been tested for HPV. Patients with recurrence were excluded, as were those with poorly preserved tissue samples, owing to the high risk of false‐negative HPV test results.

### 2.3 Sample Size

According to Gargano et al. [18], the rate of HPV infection among patients with VIC was 69%, with a median age at diagnosis of 70 years. Using a 95% confidence interval (CI), margin of error = 0.1, and *p* = 0.69, the sample size was calculated as 83 cases. A total of 95 patients with VC who fulfilled this study criteria were recruited.

### 2.4 Sample Preparation and HPV‐DNA Testing Procedure

For HPV‐DNA testing, tumor samples from each patient were prepared into formalin‐fixed, paraffin‐embedded (FFPE) tissue blocks and cut into 5–10 four‐μm slices depending on the thickness of the tumor tissue obtained. The slices were stored individually in Eppendorf tubes. To prevent HPV cross‐contamination during slicing, each blade was used for only one FFPE block, and the cutting surface was thoroughly cleaned with 70% alcohol before the next block was sliced.

The samples were processed step‐by‐step for the DNA extraction in the MBC using Promega Wizard Genomic DNA Purification Assay (Promega; Wisconsin, USA) according to the manufacturer's instructions [27]. The extracted DNA samples were tested for HPV using quantitative polymerase chain reaction (qPCR) with AccuPid HPV Genotyping Assay (KT Biotech; Ho Chi Minh City, Vietnam). This CE‐IVD (in vitro diagnostic)‐compliant assay [27, 28] can detect HPV‐16, HPV‐18, and 12 other HR‐HPVs (31, 33, 35, 39, 45, 51, 52, 56, 58, 59, 66, and 68). The HPV test results were analyzed using QuantStudio Design & Analysis, version 1.5.1 (Thermofisher Scientific; Massachusetts, USA).

### 2.5 Data Collection

Several relevant clinical and pathological characteristics were collected from the medical records and hospital electronic databases. The dependent variables were the status and type of HPV infection. The independent variables were age at diagnosis, HPV vaccination status, gravida/para/abortus (GPA), medical and sexual history, clinical stage, vulvar tumor histology, and grade. Data were input into EpiData Manager (version 4.6.0.6; Odense, Denmark) for processing and into SPSS (version 20.0; IBM Corp., Armonk, NY) for statistical analysis.

### 2.6 Statistical Analysis

The *χ* ^2^ or Fisher's test was used to test the correlation between two qualitative variables. Logistic or Poisson regression was used to test the correlation between the quantitative and binary variables. The *z*‐test was used to test the difference between the proportions of two independent studies. Statistical significance was determined using a *p*‐value < 0.05.

### 2.7 Proportional Attribution of HPV Genotypes in Co‐Infections

Cornall et al. used four different attribution algorithms to estimate lesion‐associated HPV genotype prevalence from whole tissue sections, using lesion‐specific data from laser capture microdissection (LCM) as the reference standard. The findings indicated that, when LCM is unavailable, attribution algorithms can provide a feasible approach for estimating the causative HPV genotypes in co‐infected lesions. However, researchers should be aware of the assumptions and limitations inherent in each algorithm when interpreting their results. Among these algorithms, Proportional Attribution (PropAttr) is the most often reported from studies of cervical and other gynecological lesions related to HPV [29, 30, 31].

In this study, a PropAttr model was employed to determine the most likely causative HPV genotype in those with co‐infections. Attribution estimates, computed based on mono‐infection prevalence, were validated using Spearman's rank correlation analysis to assess their alignment with observed mono‐infection prevalence. The detailed methodology was described in the Supporting Information under the subsection titled “Supplementary Analysis of the Proportional Attribution Model Approach.”

### 2.8 Ethical Considerations

Written informed consent was obtained from all participants or their legal representatives prior to their inclusion in the study. The study was approved by the Ethics Council in Biomedical Research of the Oncology Hospital, on November 4, 2020 (Approval No. 554A/BVUB‐HĐĐĐ). The study adhered to the ethical guidelines set forth by the institutional and national research committees in line with the 1964 Helsinki Declaration and its subsequent amendments. Confidentiality and anonymity of patient data were strictly maintained throughout the study.

### 2.9 The STROBE Checklist

This study was completely checked according to the STROBE checklist, which was included in the (Table S6).

## 3 Results

### 3.1 Patients' Baseline Characteristics

The study cohort was comprised of 95 individuals, with a mean age at diagnosis of 65 ± 11.8 years (95% CI: 62.3–67.2). The youngest and oldest patients were aged 39 and 94 years, respectively. The number of cases of VC gradually increased with age at diagnosis, sharply increased after the age of 50, and then declined after the age of 70.

Many patients had hypertension (50/95, > 50%), a single sexual partner (80/95, > 80%), and no family history of malignancy (93/95). Of the 63 patients for whom information was available, only one received all three doses of the HPV vaccine. Approximately one‐fifth of the patients (18/95) had other genital lesions, while less than 10% of the patients (7/95) had other primary cancers (Table 1).

**TABLE 1:** Patients' medical histories and vulvar tumor characteristics.

Characteristics (*N*)	*n* (%)	95% CI
Underlying diseases (95)		
Hypertension	50 (52.6)	43.2–63.1
Diabetes	12 (12.6)	6.3–20
Dyslipidemia	5 (5.3)	1.1–9.5
Number of sexual partners (95)		
0	1 (1.7)	—
1	80 (84.5)	75.9–93.1
2–5	14 (13.8)	5.2–22.4
Other genital lesions (95)^a^		
Yes	18 (18.9)	—
No	77 (81.1)	—
Other primary cancers (95)		
Cervix	3 (3.15)	—
Breast	3 (3.15)	—
Uterus	1 (1.1)	—
Pathohistological types (95)		
SCC‐NOS	56 (58.9)	—
kSCC	32 (33.7)	—
nkSCC	2 (2.1)	—
Adenocarcinoma	3 (3.3)	—
Basal cell carcinoma	2 (2.1)	—
Histologic grade (95)		
1	50 (52.7)	—
2	40 (41.9)	—
3	5 (5.4)	—

Abbreviations: CI, confidence interval; kSCC, keratinizing squamous cell carcinoma; nkSCC, non‐keratinizing squamous cell carcinoma; SCC‐NOS, squamous cell carcinoma‐not otherwise specified.

^a^ Regardless of being in the past or present.

In terms of VIC histology, SCC‐NOS (not otherwise specified) was the most frequent category (56/95; nearly 59%), followed by kSCC (32/95). Regarding tumor grade, the vast majority of patients were categorized as moderately differentiated (nearly 95%). Among the 94 cases for whom information was available, nearly 40% were in clinical stage I, whereas the least common was clinical stage II (nearly 10%). Among the 75 postoperative cases, clinical stage I was the most common (45%), whereas clinical stage II was the least common (12%) (Table S1).

Among the patients with other genital lesions, the most common were vulvovaginal atrophy (4.2%), genital warts (3.2%), and VIN 3 (3.2%). Other observed lesions included vulvar Paget disease (2.1%), vulvar lichen sclerosus (1.1%), vaginal intraepithelial neoplasia (VaIN) 3 (1.1%), CIN 2 (1.1%), vaginal Paget disease (1.1%), mild vulvar dysplasia (1.1%), and vulvar pigmentation (1.1%). Notably, no patient had more than one genital lesion (Table S1).

### 3.2 HPV Infection Rate and Genotypes

**FIGURE 1:**
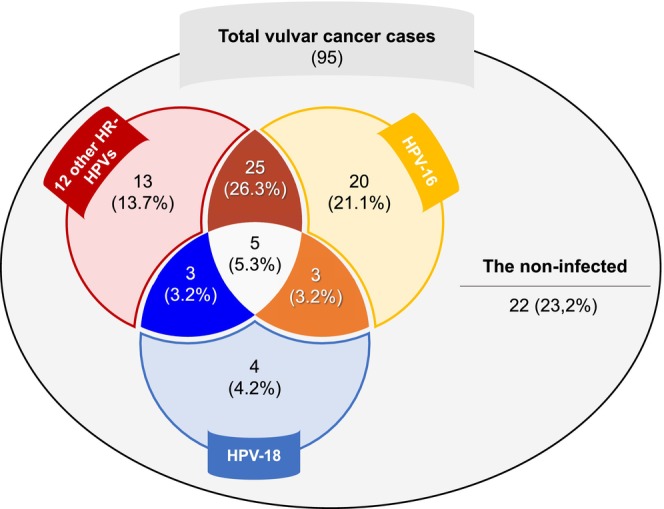
Case distribution according to the status and genotype of HPV infection. HPV, human papillomavirus; HR‐HPVs, high‐risk human papillomavirus types.

In this study, the rate of HPV infection in patients with VC was approximately 75% (73/95; 95% CI: 68.4–85.2). HPV‐16 was the most prevalent subtype, identified in more than half of the patients (53/95). Among all cases, 12 other HR‐HPVs accounted for nearly half of the cohort (46/95), whereas HPV‐18 was found in nearly 15% (15/95). The rate of HPV mono‐infection was similar to that of co‐infection, with nearly 40% of cases being irrespective of VIC or SCC. Among those with mono‐infection, HPV‐16 was the most common, found in approximately 21.1% of the VIC cases and 22.2% of the vulvar SCC cases. HPV‐18 was found in 4.2% of the VIC cases and 4.4% of the SCC cases, whereas 12 other HR‐HPVs were identified in 13.7% of the VIC cases and 14.4% of the SCC cases. Notably, triple co‐infection with HPV‐16, ‐18, and 12 other HR‐HPVs accounted for nearly 5% of all cases (Figure 1 and Table 2).

**TABLE 2:** HPV infection rate according to genotype and tumor pathohistology.

Genotype(s)	Infection rate (%)	
	VIC (*N* = 95)	Vulvar SCC (*N* = 90)
**Mono‐infection**		
16	21.1	22.2
18	4.2	4.4
12 other HR‐HPVs^a^	13.7	14.4
Total	39.0	41.0
**Co‐infection**		
16 and 18	3.2	3.3
16 and 12 other HR‐HPVs	26.3	27.8
18 and 12 other HR‐HPVs	3.2	3.3
16, 18, and 12 other HR‐HPVs	5.3	5.6
Total	38.0	40.0

Abbreviations: HPV, human papillomavirus; HR‐HPVs, high‐risk human papillomavirus types; SCC, squamous cell carcinoma; VIC, vulvar invasive carcinoma.

^a^ Patients infected with 12 other HR‐HPVs but for whom we were unable to determine their specific genotypes.

In particular, the infection rate was inversely proportional to the age at diagnosis, with a nearly 100% rate for patients aged ≤ 40 years, 90.3% for those aged 40–60 years, and 69.4% for those aged > 60 years. In terms of the lifetime number of sexual partners, the infection rate was 100% for those with 0 or 2–5 partners and 71.4% for those with a single partner. Regarding clinical stages, the highest infection rates were observed in stages I and IV (more than 80%), whereas the lowest rate was observed in stage II (approximately 40%). For vulvar pathohistological types, the rates were 77.8% for SCC, 66.7% for adenocarcinoma (AC), and 50.0% for basal cell carcinoma (BCC) (Table S2).

Regarding the PropAttr model, Spearman's correlation analysis demonstrated strong and statistically significant correlations between PropAttr estimates and mono‐infection prevalence for HPV‐16 (*ρ* = 0.806, *p* < 0.001), HPV‐18 (*ρ* = 0.992, *p* < 0.001), and the 12 other HR‐HPVs (*ρ* = 0.880, *p* < 0.001). Specifically, HPV‐16 and the 12 other HR‐HPVs showed a strong negative correlation (*ρ* = −0.751, *p* < 0.001), indicating that as HPV‐16 was assigned greater causality in co‐infections, the 12 other HR‐HPVs were less frequently attributed. A moderate negative correlation (*ρ* = −0.370, *p* = 0.001) was observed between HPV‐16 and HPV‐18, while HPV‐18 and the 12 other HR‐HPVs exhibited a weak negative correlation (*ρ* = −0.224, *p* = 0.056), which did not reach statistical significance. The detailed results are provided in the (Table S5).

**FIGURE 2:**
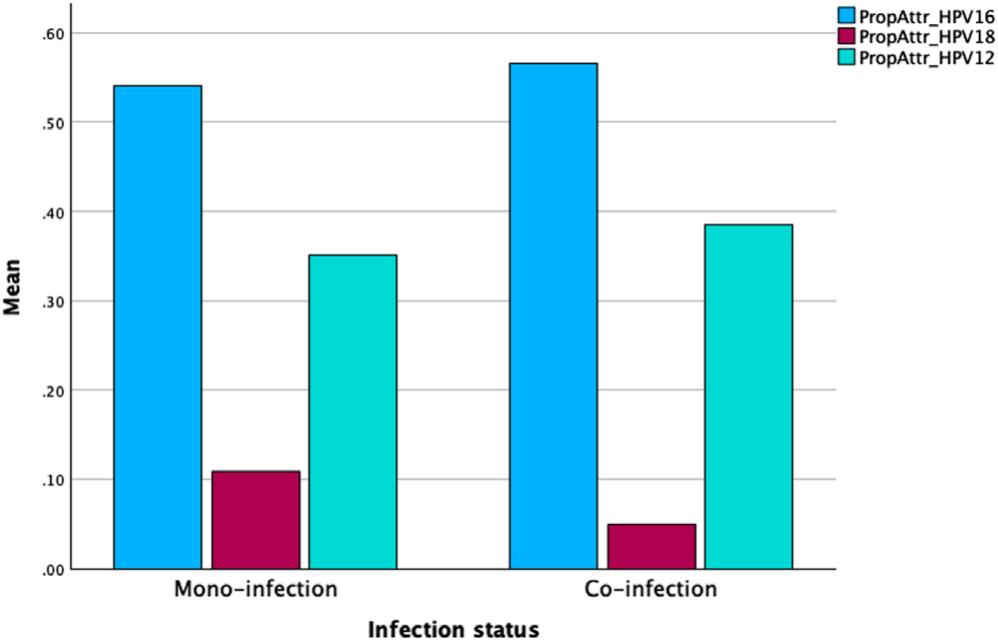
Mean proportional attribution of HPV genotypes in mono‐infections and co‐infections. PropAttr, proportional attribution.

The mean PropAttr values for HPV‐16, HPV‐18, and the 12 other HR‐HPVs were compared across mono‐infection and co‐infection, as visualized in Figure 2. Overall, HPV‐16 was the dominant genotype in both mono‐infection and co‐infection, with the highest mean PropAttr (0.55–0.60). The mean PropAttr for the 12 other HR‐HPVs ranged from 0.35 to 0.40 in both mono‐infection and co‐infection. Finally, HPV‐18 had the lowest mean PropAttr (less than 0.15).

### 3.3 HPV Infection Status and Clinicopathological Characteristics

Age at diagnosis was the only factor found to be significantly associated with HPV infection status in VC in both univariate (*p* = 0.004) and multivariate (*p* < 0.001) analyses. While the vulvar pathohistological type was significantly associated with HPV infection status in the multivariate analysis (*p* < 0.001), other factors such as the number of sexual partners and other primary cancers were statistically insignificant in both univariate and multivariate analyses (Table 3).

**TABLE 3:** Correlation between HPV infection and clinicopathological characteristics.

Characteristics	HPV infection subgroups		*p*	
	Positive	Negative	Univariate analysis	Multivariate analysis
Age at diagnosis (mean; 95% CI)	62.8 (60.1–65.4)	71.4 (66.7–76.0)	0.004**	< 0.001**
Number of sexual partners (median; 95% CI)	1,2 (1.0–1.3)	1 (SD = 0)	0.194**	0.995**
**Other primary cancers (cases)**				
Yes	7	0	0.195*	0.992**
No	66	22		
**Vulvar pathohistological types (cases)**				
SCC	70	20	0.327*	< 0.001**
AC	2	1		
BCC	1	1		

*Note:* (*) *χ* ^2^/Fisher's test; (**) Logistic regression. Bold value indicates statistially significant *p* < 0.05.

Abbreviations: AC, adenocarcinoma; BCC, basal cell carcinoma; CI, confidence interval; HPV, human papillomavirus; SCC, squamous cell carcinoma; SD, standard deviation.

### 3.4 Case Report

Among those triple co‐infected with HPV‐16, HPV‐18, and 12 other HR‐HPVs, the oldest was a female diagnosed at 69 years of age after the patient noticed a growing mass in the vulva. Physical examination revealed a firm and painless 4‐cm tumor in the right labia minora without any other genital lesions. The patient reported having more than five sexual partners in her lifetime and had a gravida/para of 7/6. The patient had never been vaccinated against HPV. Furthermore, the patient had hypertension and diabetes type II that were well controlled with treatment.

**FIGURE 3:**
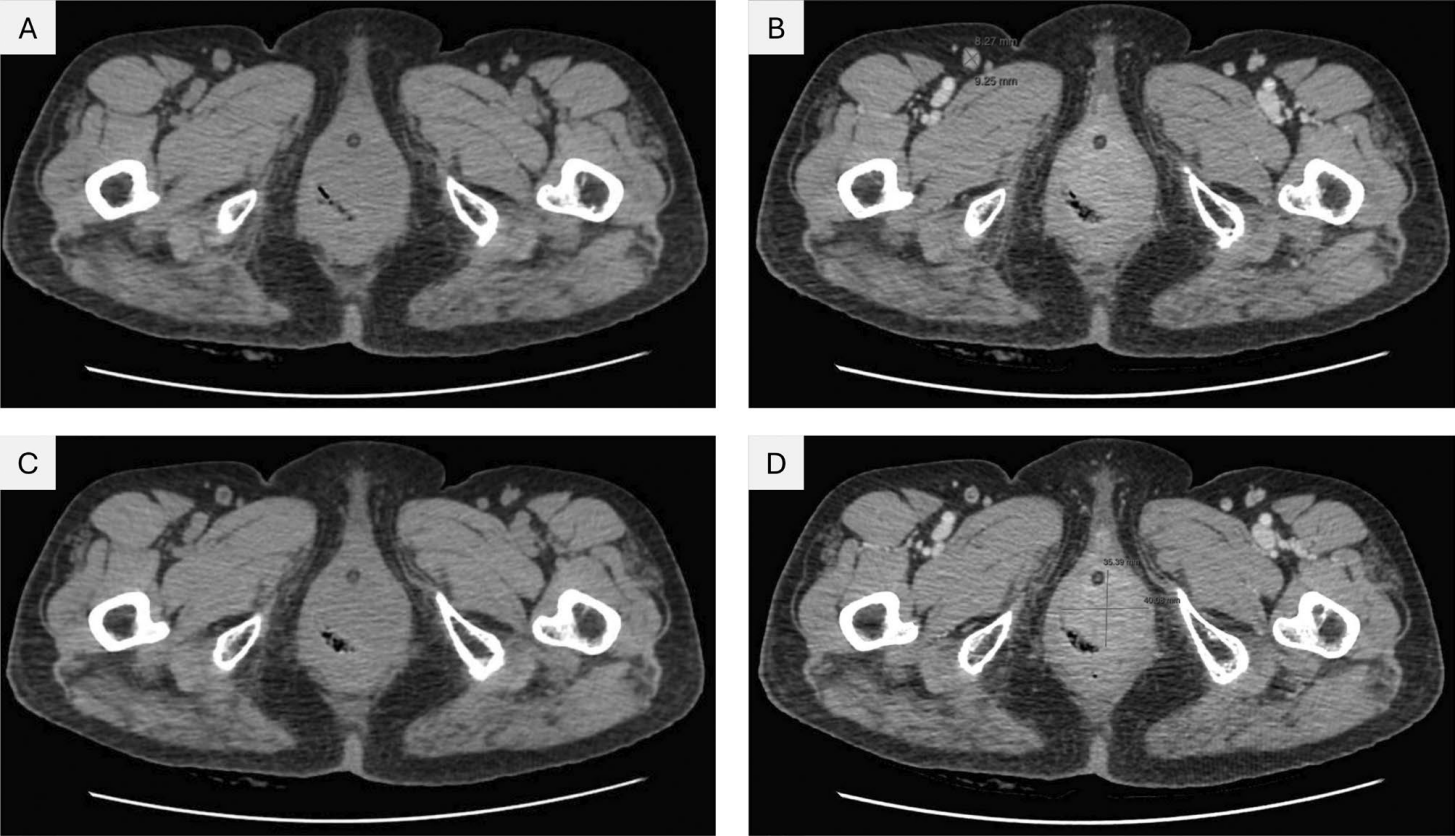
(A, B) Suspicious lymph node (LN) in the right groin on the pelvic CT scan (two crossed lines), before (left) and after (right) IV contrast injection. A well‐defined, rounded, 9.3‐mm LN with soft tissue attenuation is visible in the right groin, which is also heterogeneously and peripherally contrast‐enhanced. Overall, these features are indicative of malignancy, which was proven by an FNA‐based cytology report as LN‐metastasizing invasive carcinoma; (C, D) The labia minora solid lesion on the pelvic CT scan (two crossed lines), before (left) and after (right) IV contrast injection. This vulvar solid tumor (40 × 35 mm) is heterogeneously contrast‐enhanced and appears not to have invaded the urethra. The patient had a urethral catheter placed before undergoing CT scans to evaluate the extent of vulvar tumor invasion. CT, computed tomography; IV, intravenous; FNA, fine‐needle aspiration.

The patient underwent vulvar tumor biopsy for diagnostic evaluation. Pathological results indicated grade 2 invasive SCC. Chest, abdominal‐pelvic computed tomography (CT) scans with intravenous (IV) contrast, and bilateral neck and inguinal ultrasonography were conducted for tumor staging (Figure 3). Following the biopsy, two abnormal imaging findings were reported: (i) a rounded 9‐mm lymph node (LN) in the right groin, with fine‐needle aspiration (FNA)‐based cytology showing an invasive carcinoma, and (ii) a vulvar 4‐cm solid lesion featuring several malignant characteristics. Ultimately, the patient was diagnosed with stage III VC.

Following a comprehensive discussion between the patient, her relatives, and attending clinicians, the patient underwent definitive chemoradiotherapy (dCRT) with concurrent cisplatin (Cis) chemotherapy (40 mg/m^2^/week for 5 cycles). Based on our clinical practice protocol, external beam radiation therapy (EBRT) was prescribed for the vulvar region and bilateral pelvic and inguinal LN regions, with a total dose of 59.4 Gy (1.8 Gy per fraction). During treatment, several non‐severe acute toxicities were experienced including grade 2 genital dermatitis, grade 2 cystitis/urethritis, grade 2 neutropenia, grade 2 thrombocytopenia, grade 2 nausea, and grade 1 diarrhea, all of which were treated medically.

After completing the treatment, the patient's overall health condition was good, and the quality of life remained stable. During a total of 9 months, the patient attended three follow‐up visits after treatment. At the first visit 3 months after treatment completion, the disease showed a complete response to treatment and remained non‐recurrent as of the patient's last visit 9 months after treatment completion. Unfortunately, contact with this patient was lost after the coronavirus 2019 (COVID‐19) pandemic outbreak in 2021.

This case illustrates the occurrence of HPV‐infected VC in an elder adult patient, notably with a rare triple infection of HPV‐16, HPV‐18, and 12 other HR‐HPVs. The patient's lack of prior HPV vaccination and history of multiple sexual partners should be noted, given the elevated risk of HPV‐related cancers. Furthermore, the preventive role of HPV vaccination in reducing the risk of HPV‐associated cancers is underscored by this case. The lack of vaccination, along with HPV co‐infection, highlights the importance of widespread vaccination programs to prevent HPV‐associated cancers, including VC.

Regarding the advanced stage of the disease at the initial diagnosis in older adult women, particularly in developing countries, greater efforts are needed to encourage these women to seek medical consultation earlier, regardless of the reason. A treatment regimen comprised of dCRT with concurrent Cis demonstrated considerable efficacy in our patient. Despite the patient's advanced age and inappropriateness for curative surgery, alternative treatments were tolerated well, and the patient experienced manageable acute toxicity. At the 3‐month follow‐up, the patient exhibited a complete response to treatment, and no recurrence was noted at the 9‐month follow‐up. This suggests that dCRT is a viable alternative to curative surgery in senior patients with several underlying diseases.

## 4 Discussion

We found that the HPV infection rate was approximately 77% (68.4–85.2) in VC patients, with HPV‐16 being the most common subtype (nearly 56%). Patients infected with HPV (mean age: 62.8) were younger than those who were not infected (mean age: 71.4) in both univariate (*p* = 0.004) and multivariate (*p* < 0.001) analyses. The VC HPV infection rate in Vietnam was significantly higher than that in other Asian countries. This implies the considerable health burden of HPV‐related VC. Our results highlight the importance of HPV vaccines covering HPV‐16, particularly in young women.

### 4.1 Patients' Baseline Characteristics

In this study, the average age of diagnosis was 64.8 years (95% CI: 62.3–67.2) with the VC diagnosis rate increasing with age, similar to previous findings reported by Siriaunkgul et al. [32] and Hampl et al. [33] More than half of the patients had hypertension, significantly higher than the nearly 13% reported by Xiao et al. (*p* < 0.001), possibly due to the older patient group (64.9 vs. 51.9 years). The rate of diabetes was similar to that reported by Xiao et al. (*p* = 0.41) [24]. Of the 44 patients with the available relevant information, the majority had one sexual partner (nearly 70%), while the remaining group had two to five sexual partners. This is likely underreported due to cultural sensitivities in Asia [34]. Of the 63 patients with records of vaccinations, only one received all three doses of the HPV vaccine. This result is not surprising, as the HPV vaccine was not licensed in Vietnam until 2007, by which time most patients were beyond the recommended age for vaccination [35, 36, 37]. Nearly one‐fifth of the patients in this study had other genital lesions, significantly lower than that reported in previous reports (*p* < 0.001) [18, 24, 38, 39]. This difference may be due to underdiagnosis or underreporting, likely due to the disadvantages of retrospective research (e.g., recall bias and sample selection) rather than the pathological nature of VC. The rates of other primary cancers in our study were similar to those reported by Xiao et al. [24].

### 4.2 HPV Infection Rate

This study's HPV infection rate was similar to that reported by Gargano et al. (*p* = 0.17) and Sutton et al. (*p* = 0.2) [18, 40], but significantly higher than that published by authors reporting on European and Asian populations (*p* ≤ 0.001), as detailed in the (Tables S3 and S4) [23, 24, 39, 41]. According to medical literature, this rate fluctuates considerably according to geographical region, without clear reasons [12, 13]. In general, potential factors can be categorized into two groups: technical (e.g., testing method, specific assay, sample preparation, sample preservation quality, and specimen type) and biological (e.g., sexual behavior, HPV vaccination, smoking status, sexually transmitted diseases, and genetic factors) [12, 13].

First, the sample storage duration and preservation conditions could lead to false‐negative (FN) results [42, 43, 44], due to the degradation of sample DNA prior to testing [45]. This study had a short storage duration (1–2 years) compared to that of previous studies (3–21 years), which may have increased the FN risk [23, 24, 35, 36, 39, 41]. However, Gargano et al. and Sutton et al. reported an infection rate similar to that of this study (*p* > 0.05), despite their long storage duration, indicating that other factors may be responsible, such as the use of specific testing methods or assays. For instance, Gargano et al. sequentially employed the linear array HPV and the INNO‐LiPA HPV genotyping assay, before noting their sample as truly negative [18]. Thus, the sensitivity improved by this special approach may compensate for the FN risk due to the long storage duration. As per Sutton et al., the cross‐infection risk cannot be ruled out, since their sample preparation procedure was not described clearly [40]. Other factors, such as the thickness and quantity of the sample slices used for HPV testing, have yet to be agreed upon [45]. HPV test assays also vary widely among different studies [23, 24, 35, 36, 39, 41]. Overall, these authors used the assays that were considerably compatible (> 90%) with standard methods (e.g., PCR, electrophoresis, and gene sequencing). In this study, the AccuPid HPV genotyping assay certified by the CE‐IVD was employed [28, 46, 47]. Thus, differences in HPV infection rates are unlikely to have resulted from differences in the HPV assays used, although these compatibility comparisons are mostly indirect.

According to the literature, the European population is less likely to be infected with HPV than Asian and North American populations [12, 13]. Similarly, this study's population had a significantly higher infection rate than the European population (*p* ≤ 0.001) [23, 39, 41]. Regarding VC pathology, warty and basal SCC are considered closely related to HPV, as shown by Sutton et al. and Gargano et al., who reported higher infection rates. In these studies, the warty and basal SCC subgroups accounted for nearly one‐third of all cases [18, 40]. This consistency supports the reported high HPV infection rates. Given the similarity in infection rates between this study and those reported by these authors, we believe that the warty and basal SCC subgroups accounted for a considerable proportion of cases in this study; however, they were likely underdiagnosed as SCC‐not otherwise specified (NOS).

Furthermore, age at diagnosis was mostly attributed to differences in HPV infection rates among different studies [1, 14, 18, 24, 32, 40, 48]. Unfortunately, we were unable to investigate this factor among the studies because of the incomparability of the reported data [18, 23, 24, 25, 40, 41]. As reported for CC, the number of sexual partners and sexually transmitted diseases may be associated with HPV infection risk; however, these are usually difficult to survey retrospectively [12, 49, 50, 51, 52]. Additionally, differences in genetic characteristics could also be attributed to variations in HPV infection risk and clearance, thereby indirectly affecting the infection rates among the retrospective studies [18, 40].

### 4.3 Infection Genotypes

Consistent with previous reports, we found HPV‐16 to be the most common type of HPV in patients with VC [18, 23, 25, 39, 40, 41]. This suggests that HPV‐16, known as the strongest oncogenic type among HR‐HPVs, remains the primary etiology among HPV‐related cancers. The prevalence of HPV‐16 in different cancers may be attributed to similarities in the HPV‐16‐related oncogenic pathway and prevention strategies. Notably, as more than half of the patients in this study were infected with HPV‐16, considerable preventive benefits can be expected from vaccination, as many current vaccines cover this type of HPV. From a clinical perspective, even when access to the 9‐valent vaccine is limited due to economic barriers, particularly in developing countries, the 2‐valent and 4‐valent vaccines remain highly effective in preventing VC. As demonstrated in this study, the 2‐valent and 4‐valent vaccines could help prevent nearly 60% of the total HPV‐16‐related cancers and nearly 70% of HPV‐16 and HPV‐18‐related cancers collectively.

We also found that 12 other HR‐HPVs were more common than HPV‐18. As the AccuPid partial genotyping assay combined these 12 other HR‐HPVs, the collective rate was higher than that of HPV‐18 alone. Overall, the rate of HPV‐18 infection in this study was significantly higher than that reported previously (*p* < 0.05). Additionally, the most common type of HPV after HPV‐16 in patients with VC remains inconsistent across studies and has been reported to be HPV‐33 [12, 18, 25, 40, 41, 53], HPV‐18 [39, 54], or HPV‐89 [32]. In this study, an HR‐HPV co‐infection rate of approximately 40% was observed, which is significantly higher than that reported by Gargano et al. (5.6%, *p* < 0.001) and Sutton et al. (10.3%, *p* < 0.001). The factors contributing to the co‐infection rate remain unelucidated; however, many authors have considered geographic region, ethnicity, and genetic factors [40]. The most common form of co‐infection involved HPV‐16, similar to the findings of other studies, suggesting that HPV‐16 has the strongest oncogenic potential in VC, irrespective of whether it is a mono‐infection or co‐infection [18, 40].

Cornall et al. demonstrated that attribution algorithms can yield results comparable to those obtained through LCM when properly validated [29]. According to the PropAttr model applied in our study, strong positive correlations between PropAttr estimates and mono‐infection prevalence (HPV‐16: *ρ* = 0.806, *p* < 0.001; HPV‐18: *ρ* = 0.992, *p* < 0.001; 12 other HR‐HPVs: *ρ* = 0.880, *p* < 0.001) suggest that this model is reliable for attributing HPV genotypes in co‐infected cases and effectively captures the real‐world distribution of HPV genotypes in VC lesions.

The consistently high PropAttr of HPV‐16 across both mono‐infections and co‐infections supports its well‐established dominance in vulvar carcinogenesis [12, 13]. This reinforces the biological validity of our dataset, as HPV‐16 is the most oncogenic HR‐HPV globally. The stable PropAttr of the 12 other HR‐HPVs across both mono‐infections and co‐infections (0.35–0.40) further suggests that these HR‐HPVs are being accurately classified, without significantly distorting the overall prevalence estimates of HR‐HPVs.

Additionally, this model identified negative correlations between PropAttr estimates of different HR‐HPVs, indicating competitive assignment patterns within co‐infections. The strong negative correlation between HPV‐16 and the 12 other HR‐HPVs (*ρ* = −0.751, *p* < 0.001) suggests that HPV‐16 is more likely to be assigned in VC lesions, while the 12 other HR‐HPVs are less frequently attributed in such cases. While HPV‐18 is classified as a HR genotype, it is less frequently attributed in lesions where HPV‐16 is present, suggesting a preferential dominance of HPV‐16 in co‐infections. These findings align with previous research in VC, confirming HPV‐16's central role in vulvar carcinogenesis and its competitive interaction with other HR‐HPVs [12, 13].

### 4.4 Clinicopathologic Characteristics and HPV Infection Status

Age at diagnosis was the only factor significantly associated with HPV infection in patients with VC in both the univariate and multivariate analyses. Similar to previous reports, the HPV infection rate in VC declined with age [32, 53, 55, 56]. In this study, the HPV‐infected group was approximately 10 years younger than the uninfected group (62.8 vs. 71.4 years). This illustrates the phenomenon of VC diagnosis at a younger age in recent decades, which is widely believed to result from an increase in the rate of HPV infection among young women [1, 2, 48].

The vulvar pathohistological type was found to be significantly associated with HPV infection status in the multivariate analysis only. This suggests that HPV infection is highly likely in patients with SCC VC, rather than in non‐SCC VC, such as AD and BCC, regardless of age and clinical stage. As discussed, we believe that the warty and basal SCC subgroups accounted for a considerable proportion of cases in this study, but were likely underdiagnosed as SCC‐NOS. This is also perceived as a limitation of this study, as we were unable to clearly identify specific subtypes of SCC‐NOS.

Although other factors did not show statistical significance, this may be due to several limitations of this study, such as the sample size, selection bias, and recall bias. Another limitation was that the HPV genotyping assay is unable to detect the specific type(s) of the 12 other HR‐HPVs subgroups. Thus, we were unable to determine which types of HPV other than HPV‐16 and HPV‐18 were present in the patients with VC [18, 23, 24, 39, 40, 41].

### 4.5 HPV Vaccination Strategy

Despite the discovery of HPV as an etiology and the demonstrated benefits of HPV vaccination in CC and several external genital lesions [57, 58, 59], vaccination coverage remains low in many developing countries, regardless of the availability and use of a 1‐dose regimen or regimens with more doses. This phenomenon may result from a variety of barriers, such as unaffordability due to the high cost of the HPV vaccine, lack of healthcare awareness, and lack of access to healthcare, regardless of need and readiness [9, 26].

As reported, the HPV infection rate in patients with VC in Vietnam is significantly higher than that in other Asian countries, reflecting a considerable health burden caused by HPV that is above that of other regional countries. This strongly implies the effectiveness of HPV vaccines covering HPV‐16 and HPV‐18 for VC prevention (approximately 70%), particularly in young women. Thus, the implementation of an HPV vaccination strategy based on the infection rate in patients with VC could provide tangible benefits for women in Vietnam.

### 4.6 The Limitations

We acknowledge that a simple HPV DNA test cannot differentiate between bystander and transforming HPV infections, where viral oncogenes disrupt the cell cycle [8, 60, 61]. Reuschenbach et al. provide critical evidence regarding the role of p16INK4a and p53 IHC in VC, demonstrating the tumorigenic effects of HPV in these malignancies [39]. At the time this study was conducted, IHC was not performed due to limited personnel, complex reagent cost calculations, and the COVID‐19 outbreak in 2022, which disrupted reagent supplies and prevented external sample testing. Thus, the absence of these data partially limits the interpretation of our results. In future studies, we plan to perform p16INK4a and p53 IHC staining alongside HPV DNA testing to enhance the accuracy and depth of our findings.

Another limitation is the absence of additional contamination control measures, such as decontaminating cutting surfaces with an RNA/DNA removal solution or incorporating positive and negative controls, which may have affected the accuracy of HPV detection and increased the risk of false‐positive results. To mitigate cross‐contamination, each blade was used for a single FFPE block, and the cutting surface was thoroughly cleaned with 70% alcohol before processing the next sample in our study. The high co‐infection rate observed in our study (approximately 40%), however, still raises the possibility of carryover contamination during sample processing. Thus, the lack of more rigorous decontamination protocols remains a constraint, potentially influencing the reliability of the findings.

The lack of access to laser microdissection, an advanced technique that enables precise isolation of individual lesions for HPV typing, is another limitation in this study. As Quint et al. found [62], each lesion is typically clonal, containing a single HPV subtype. Thus, the high proportion of multiple HPV infections reported in this study should be interpreted with caution, as it may be influenced by technical limitations rather than true co‐infection at the lesion level. In our study, although the PropAttr model applied for providing a statistically validated approach for estimating HPV genotype causality in co‐infected lesions, we also acknowledge several limitations of this model.

The PropAttr model assumes that the prevalence of HPV types in mono‐infection reflects their oncogenic potential in co‐infections. However, HPV genotypes may interact in ways that alter their relative carcinogenic risk, which this approach does not capture. A study found certain HPV subtypes may synergistically enhance oncogenic potential in co‐infections, leading to underestimation or overestimation of specific genotypes [63]. In addition, this model does not account for viral load, which could be a crucial factor in determining which HPV type is driving lesion progression. Studies using viral load‐based attribution models have suggested that the HPV genotype with the highest viral load in a lesion is the most likely causal agent [64, 65]. Therefore, integrating viral load data and machine learning approaches may further enhance accuracy in HPV genotype assignment for oncogenic risk stratification in future studies.

## 5 Conclusions

In this study, the HPV infection rate among Vietnamese patients with VC was significantly higher than previously reported in other Asian populations, indicating a substantial disease burden driven primarily by HPV‐16 and HPV‐18. The strong association between younger age at diagnosis and HPV infection status highlights the importance of early HPV exposure in disease development and reinforces the need for targeted preventive strategies. Our findings underscore the urgent need for national HPV vaccination programs, particularly in young women, to mitigate the risk of HPV‐associated VC. Future studies should incorporate viral load‐based attribution models, LCM techniques, and machine learning‐based approaches to improve accuracy in oncogenic risk stratification and HPV genotype causality assessment.

## Author Contributions

D.N.L.T.: conceptualized and designed the study, collected and analyzed the data, and drafted the manuscript. H.N.V.: contributed to data analysis, interpretation, and manuscript revision. T.T.A.C.: assisted with data validation and methodological support. N.D.T.: and H.N.C.: supervised the research, provided critical insights, secured funding, and approved the final version of the manuscript. All authors reviewed and approved the final manuscript.

## Ethics Statement

Written informed consent was obtained from all participants or their legal representatives prior to their inclusion in the study. The study was approved by the Ethics Council in Biomedical Research of the Oncology Hospital, on November 4, 2020 (Approval No. 554A/BVUB‐HĐĐĐ). The study adhered to the ethical guidelines set forth by the institutional and national research committees in line with the 1964 Helsinki Declaration and its subsequent amendments. Confidentiality and anonymity of patient data were strictly maintained throughout the study.

## Conflicts of Interest

The authors declare no conflicts of interest.

## Supporting information


**Data S1.** Supplementary analysis of the proportional attribution model approach.
**Table S1.** Other genital lesion types among cohort. CIN, cervical intraepithelial neoplasia; VaIN, vaginal intraepithelial neoplasia; VIN, vulvar intraepithelial neoplasia.
**Table S2.** HPV infection rate according to several different subgroups. AC, adenocarcinoma; BCC, basal cell carcinoma; HPV, human papillomavirus; SCC, squamous cell carcinoma.
**Table S3.** Technical factors that could impact the rate of HPV infection. FFPE, formalin‐fixed paraffin‐embedded; HPV, human papillomavirus; qPCR, qualitative polymerase chain reaction; RLBH, reverse line blot hybridization.
**Table S4.** Biological factors that could impact the rate of HPV infection. CI, confidence interval; HPV, human papillomavirus; IQR, interquartile range; kSCC, keratinizing squamous cell carcinoma; nkSCC, non‐keratinizing squamous cell carcinoma; NOS, not otherwise specified; SCC, squamous cell carcinoma.
**Table S5.** Spearman’s rank correlation between proportional attribution estimates and mono‐infection prevalence of HPV genotypes.
**Table S6.** The STROBE checklist of items that should be included in reports of cross‐sectional studies.

## Data Availability

The data from this study are available from the corresponding author upon reasonable request.
