# Supplementary material for: High Prevalence of Human Papillomavirus in Vulvar Cancer Among Vietnamese Women: Implications for Vaccination Strategies
Source: Cancer Med. 2025 Jun 5;14(11):e70982. doi: 10.1002/cam4.70982 (PMC12138206; doi:10.1002/cam4.70982)
Supplement: Supplementary file 1 — Data S1. Supplementary analysis of the proportional attribution model approach. Table S1. Other genital lesion types among cohort. CIN, cervical intraepithelial neoplasia; VaIN, vaginal intraepithelial neoplasia; VIN, vulvar intraepithelial neoplasia. Table S2. HPV infection rate according to several different subgroups. AC, adenocarcinoma; BCC, basal cell carcinoma; HPV, human papillomavirus; SCC, squamous cell carcinoma. Table S3. Technical factors that could impact the rate of HPV infection. FFPE, formalin‐fixed paraffin‐embedded; HPV, human papillomavirus; qPCR, qualitative polymerase chain reaction; RLBH, reverse line blot hybridization. Table S4. Biological factors that could impact the rate of HPV infection. CI, confidence interval; HPV, human papillomavirus; IQR, interquartile range; kSCC, keratinizing squamous cell carcinoma; nkSCC, non‐keratinizing squamous cell carcinoma; NOS, not otherwise specified; SCC, squamous cell carcinoma. Table S5. Spearman’s rank correlation between proportional attribution estimates and mono‐infection prevalence of HPV genotypes. Table S6. The STROBE checklist of items that should be included in reports of cross‐sectional studies. [file CAM4-14-e70982-s001.docx]

**Supplemental Materials**

**Supplementary Analysis of the Proportional Attribution Model Approach**

1. **Dataset Preparation and Categorization**

**Infection Classification:** Each sample was classified as either a mono-infection or a co-infection (two or more HPV genotypes detected).

**HPV Genotype Variables:** Binary variables (1 = present, 0 = absent) were created for each HPV genotype (e.g., HPV-16, HPV-18, 12 other HR-HPVs).

**Defining the Attribution Reference Set:** Only cases with mono-infection were used to calculate genotype prevalence.

1. **Calculation of Mono-Infection Prevalence**

To establish a baseline reference for attribution, the prevalence of each HPV genotype among mono-infection cases was calculated as:


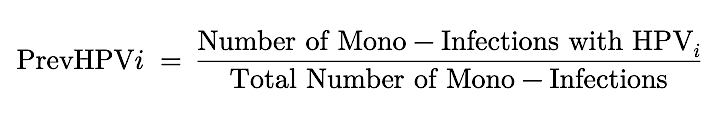


(**PrevHPVi** represents the prevalence of HPV genotype i *(HPVi)* in lesions with mono-infection)

1. **Assignment of Proportional Attribution Scores**

For each sample with a co-infection, a proportional weight was assigned to each detected HPV genotype, based on its mono-infection prevalence. This approach ensures that genotypes with higher prevalence in mono-infections are assigned greater causality in co-infections, reflecting their likely oncogenic role. The formula used was:

**
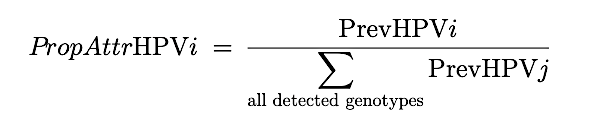
**

**(PropAttr_HPVi_** represents the proportional-attribution score for HPV genotype i; **PrevHPVi** is its prevalence in mono-infection; **and the denominator** is the sum of mono-infection prevalences for all HPV genotypes identified in the same lesion.)

1. **Validation of Attribution Model**

To assess the accuracy of the Proportional Attribution model, we conducted Spearman’s rank correlation analysis between Proportional Attribution estimates **(PropAttr_HPVi_)** and Observed mono-infection prevalence rates **(PrevHPVi)**. Strong positive correlations would confirm the reliability of the attribution model in approximating genotype-specific causality in lesions.

**Table S1.** Other genital lesion types among cohort

| **Other genital lesion types (N=95)** | **n* (%)** |
| --- | --- |
| Vaginal-vulvar atrophy | 4 (4.2) |
| Genital warts | 3 (3.2) |
| VIN 3 | 3 (3.2) |
| Vulvar Paget’s disease | 2 (2.1) |
| Vulvar lichen sclerosus | 1 (1.1) |
| VaIN 3 | 1 (1.1) |
| CIN 2 | 1 (1.1) |
| Vaginal Paget’s disease | 1 (1.1) |
| Vulvar mild dysplasia | 1 (1.1) |
| Vulvar pigmentation | 1 (1.1) |

(*) None of these cases had more than one other genital lesion type

VIN, vulvar intraepithelial neoplasia; VaIN, vaginal intraepithelial neoplasia; CIN, cervical intraepithelial neoplasia

**Table S2.** HPV infection rate according to several different subgroups

| **Characteristics (N)** | | **Infection rate (%)** |
| --- | --- | --- |
| *Age at diagnosis (95)* | ≤ 40 (2) | 100.0 |
|  | > 40 and ≤ 60 (31) | 90.3 |
|  | > 60 (62) | 69.4 |
| *Number of sexual partners (44)* | 0 (1) | 100.0 |
|  | 1 (35) | 71.4 |
|  | 2-5 (8) | 100.0 |
| *Clinical stage (71)* | I (30) | 81.1 |
|  | II (4) | 40.0 |
|  | III (22) | 75.9 |
|  | IV (15) | 83.3 |
| *Vulvar pathohistological types (95)* | SCC (90) | 77.8 |
|  | AC (3) | 66.7 |
|  | BCC (2) | 50.0 |

HPV, human papillomavirus; SCC, squamous cell carcinoma; AC, adenocarcinoma; BCC, basal cell carcinoma

**Table S3.** Technical factors that could impact the rate of HPV infection

| **Authors** | **HPV infection rate (%)** | **Publication year ^[[1]](#footnote-1)^** | **Diagnosis year ^[[2]](#footnote-2)^** | **Methods and/or assays** | **Cross-contamination prevention methods** |
| --- | --- | --- | --- | --- | --- |
| Villadsen et al.^1^ | 41.1 | 2021 | 2010–2012 | PCR, electrophoresis, and gene sequencing | Replaced the blade, sanitized the cutting surface with alcohol and RNase-away solution, and used the paraffin block without specimen as a negative control |
| Gargano et al.^2^ | 69.8 | 2012 | 1995–2005 | PCR & RLBH/ Linear Array HPV Genotyping Assay (Roche Diagnostics, USA)  Reverse hybrid technique/INNO-LiPA HPV Genotyping Assay (Innogenetics, Belgium) | Replaced the blade and sanitized the cutting surface |
| Sutton et al.^3^ | 69.8 | 2008 | 1987–2007 | PCR & RLBH/ Linear Array HPV Genotyping Assay (Roche Diagnostics, USA) | Yes, but not clearly described |
| Xiao et al.^4^ | 44.2 | 2017 | 2006–2016 | Unknown | |
| This study | 77.8 | 2022 | 2020–2021 | qPCR/ AccuPid HPV Genotyping Assay (Khoa Thuong, Vietnam) | Replaced the blade and sanitized the cutting surface with 70 % alcohol |

HPV, human papillomavirus; qPCR, quantitative polymerase chain reaction; RLBH, reverse line blot hybridization; FFPE, formalin-fixed paraffin-embedded

**Table S4.** Biological factors that could impact the rate of HPV infection

| **Authors** | **HPV infection rate (%)** | **Race** | **Age at diagnosis (95% CI)** | **SCC pathological subtypes (%)** | | | | |
| --- | --- | --- | --- | --- | --- | --- | --- | --- |
|  |  |  |  | **NOS** | **kSCC** | **nkSCC** | **Warty SCC** | **Basal SCC** |
| Villadsen et al.^1^ | 41.1 | Caucasian | 69 (IQR: 57–79) | 0 | 82.1 | 17.9 | 0 | 0 |
| Wakeham et al.^5^ | 52.0 |  | 65 (IQR: 50–78) | - | | | | |
| Reuschenbach et al.^6^ | 43.7 |  | (-) | - | | | | |
| Gargano et al.^2^ | 69.8 | Non-Hispanic White | 70 | 0 | 35.0 | 8.9 | 35.0 | 16.6 |
|  |  |  | - |  |  |  | 4.5 | |
| Sutton et al.^3^ | 69.8 |  | 66 | 61.2 | 0 | 0 | 30.2 | 8.6 |
| Xiao et al.^4^ | 44.2 | Asian | 51.9 (35.9–67.9) | - | | | | |
| Ngamkham et al.^7^ | 46.6 |  | (-) | 100 | 0 | | | |
| This study | 77.8 |  | 64.9 (62.5–67.4) | 63.3 | 35.2 | 1.5 | 0 | |

(-) No information from the original article. HPV, human papillomavirus; CI, confidence interval; IQR, interquartile range; SCC, squamous cell carcinoma; NOS, not otherwise specified; kSCC, keratinizing squamous cell carcinoma; nkSCC, non-keratinizing squamous cell carcinoma

**Table S5.** Spearman’s rank correlation between Proportional Attribution estimates and mono-infection prevalence of HPV genotypes.

**
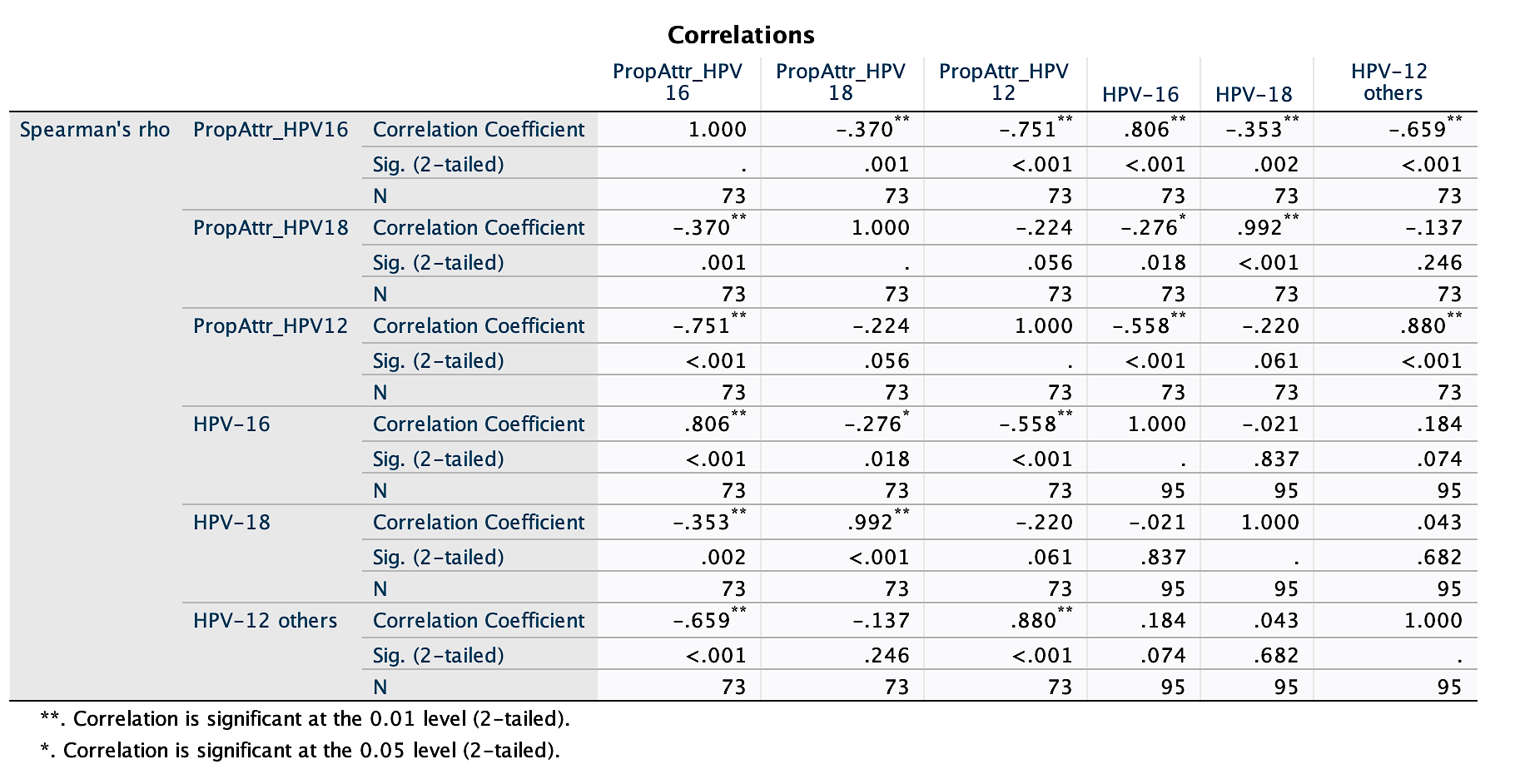
**

Spearman’s correlation coefficients (ρ) assess the relationship between Proportional Attribution (PropAttr) estimates (PropAttr_HPV16, PropAttr_HPV18, PropAttr_HPV12) and mono-infection prevalence rates of HPV genotypes (HPV-16, HPV-18, and 12 other HR-HPVs). Strong positive correlations (ρ > 0.7, p < 0.01) between attribution estimates and mono-infection prevalence confirm the reliability of the PropAttr model. Negative correlations between HPV attribution values suggest competition in genotype assignment within co-infections. Statistically significant correlations are marked as p < 0.05 (*) and p < 0.01 (**).

**References**

1. Brusen Villadsen A., Bundgaard-Nielsen C., Ambühl L., Tang Svendsen M., Søkilde Pedersen I., Stæhr Hansen E.*, et al*. Prevalence and type distribution of human papillomavirus infections in Danish patients diagnosed with vulvar squamous cell tumors and precursors. *Gynecologic oncology reports*. Aug 2021;37:100828. doi:10.1016/j.gore.2021.100828

2. Gargano J.W., Wilkinson E.J., Unger E.R., Steinau M., Watson M., Huang Y.*, et al*. Prevalence of human papillomavirus types in invasive vulvar cancers and vulvar intraepithelial neoplasia 3 in the United States before vaccine introduction. *Journal of lower genital tract disease*. Oct 2012;16(4):471-9. doi:10.1097/LGT.0b013e3182472947

3. Sutton B.C., Allen R.A., Moore W.E., Dunn S.T. Distribution of human papillomavirus genotypes in invasive squamous carcinoma of the vulva. *Modern pathology : an official journal of the United States and Canadian Academy of Pathology, Inc*. Mar 2008;21(3):345-54. doi:10.1038/modpathol.3801010

4. Xiao X., Meng Y.-B., Bai P., Zou J., Zhang Y., Nguyen T.M.B.*, et al*. Vulvar Cancer in China: Epidemiological Features and Risk Analysis. *J Cancer*. 2017;8(15):2950-2958. doi:10.7150/jca.20496

5. Wakeham K., Kavanagh K., Cuschieri K., Millan D., Pollock K.G., Bell S.*, et al*. HPV status and favourable outcome in vulvar squamous cancer. *International journal of cancer*. Mar 1 2017;140(5):1134-1146. doi:10.1002/ijc.30523

6. Reuschenbach M., Roos J., Panayotopoulos D., Baldus S.E., Schnürch H.G., Berger A.*, et al*. Characterization of squamous cell cancers of the vulvar anterior fourchette by human papillomavirus, p16INK4a, and p53. *Journal of lower genital tract disease*. Jul 2013;17(3):289-97. doi:10.1097/LGT.0b013e31826f2b2b

7. Ngamkham J., Boonmark K., Phansri T. Detection and Type-Distribution of Human Papillomavirus in Vulva and Vaginal Abnormal Cytology Lesions and Cancer Tissues from Thai Women. *Asian Pacific journal of cancer prevention : APJCP*. 2016;17(3):1129-34. doi:10.7314/apjcp.2016.17.3.1129

**Table S6.** The STROBE checklist of items that should be included in reports of cross-sectional studies

|  | Item No. | Recommendation | Page No. |
| --- | --- | --- | --- |
| **Title and abstract** | 1 | (*a*) Indicate the study’s design with a commonly used term in the title or the abstract | 1-2 |
|  |  | (*b*) Provide in the abstract an informative and balanced summary of what was done and what was found | 3 |
| Introduction | | | |
| Background/rationale | 2 | Explain the scientific background and rationale for the investigation being reported | 4-5 |
| Objectives | 3 | State specific objectives, including any prespecified hypotheses | 5 |
| **Methods** | | | |
| Study design | 4 | Present key elements of study design early in the paper | 5 |
| Setting | 5 | Describe the setting, locations, and relevant dates, including periods of recruitment, exposure, follow-up, and data collection | 5-7 |
| Participants | 6 | (*a*) Give the eligibility criteria, and the sources and methods of selection of participants | 5-6 |
| Variables | 7 | Clearly define all outcomes, exposures, predictors, potential confounders, and effect modifiers. Give diagnostic criteria, if applicable | 6-7 |
| Data sources/ measurement | 8* | For each variable of interest, give sources of data and details of methods of assessment (measurement). Describe comparability of assessment methods if there is more than one group | 6-7 |
| Bias | 9 | Describe any efforts to address potential sources of bias | - |
| Study size | 10 | Explain how the study size was arrived at | 6 |
| Quantitative variables | 11 | Explain how quantitative variables were handled in the analyses. If applicable, describe which groupings were chosen and why | - |
| Statistical methods | 12 | (*a*) Describe all statistical methods, including those used to control for confounding | 7 |
|  |  | (*b*) Describe any methods used to examine subgroups and interactions | 7 |
|  |  | (*c*) Explain how missing data were addressed | - |
|  |  | (*d*) If applicable, describe analytical methods taking account of sampling strategy | 6 |
|  |  | (*e*) Describe any sensitivity analyses | - |
| Results | | | |
| Participants | 13* | (a) Report numbers of individuals at each stage of study—eg numbers potentially eligible, examined for eligibility, confirmed eligible, included in the study, completing follow-up, and analysed | 7-8 |
|  |  | (b) Give reasons for non-participation at each stage | - |
|  |  | (c) Consider use of a flow diagram | - |
| Descriptive data | 14* | (a) Give characteristics of study participants (eg demographic, clinical, social) and information on exposures and potential confounders | 7-8 |
|  |  | (b) Indicate number of participants with missing data for each variable of interest | 23-24 |
| Outcome data | 15* | Report numbers of outcome events or summary measures | 8-9 |
| Main results | 16 | (*a*) Give unadjusted estimates and, if applicable, confounder-adjusted estimates and their precision (eg, 95% confidence interval). Make clear which confounders were adjusted for and why they were included | 12-15  24-25 |
|  |  | (*b*) Report category boundaries when continuous variables were categorized | 7 |
|  |  | (*c*) If relevant, consider translating estimates of relative risk into absolute risk for a meaningful time period | - |
| Other analyses | 17 | Report other analyses done—eg analyses of subgroups and interactions, and sensitivity analyses | - |
| **Discussion** | | | |
| Key results | 18 | Summarise key results with reference to study objectives | 11 |
| Limitations | 19 | Discuss limitations of the study, considering sources of potential bias or imprecision. Discuss both direction and magnitude of any potential bias | 13-14 |
| Interpretation | 20 | Give a cautious overall interpretation of results considering objectives, limitations, multiplicity of analyses, results from similar studies, and other relevant evidence | 12-15  Table S3-S4 |
| Generalisability | 21 | Discuss the generalisability (external validity) of the study results | 16 |
| Other information | | | |
| Funding | 22 | Give the source of funding and the role of the funders for the present study and, if applicable, for the original study on which the present article is based | 17 |

*Give information separately for exposed and unexposed groups.

1. “Publication year” relatively denotes the year in which the specimens were tested with an HPV assay. [↑](#footnote-ref-1)
2. “Diagnosis year” relatively denotes the year in which the specimens were archived as FFPE blocks as at the time the patients were diagnosed with vulvar cancer. [↑](#footnote-ref-2)
